# Single-Electron Bond in Ir–Ir Dimer Stabilized under Pressure

**DOI:** 10.1021/acs.inorgchem.5c04558

**Published:** 2025-11-11

**Authors:** Cheng Peng, Mingyu Xu, Jie Li, Weiwei Xie

**Affiliations:** † Department of Chemistry, 3078 Michigan State University, East Lansing, Michigan 48824, USA; ‡ Department of Earth and Environmental Sciences, University of Michigan, Ann Arbor, Michigan 48109, USA

## Abstract

Odd-electron bonds, often considered ephemerally stable in chemical systems, can give rise to exotic physical phenomena in quantum materials. In this study, we present the design and discovery of a new iridate, Ba_3_NbIr_2_O_9_, which hosts a single-electron bond within an Ir–Ir dimer. Ba_3_NbIr_2_O_9_ was synthesized under high-pressure and high-temperature conditions and crystallizes in a hexagonal structure (space group *P*6_3_/*mmc*). The Ir–Ir (III, IV) dimer contains an unpaired electron, leading to an electronically unstable state with potentially frustrated magnetic interactions. Magnetic susceptibility measurements reveal a paramagnetic ground state with no long-range magnetic order down to 1.8 K, despite a Curie–Weiss temperature of −26.9 K. A sign of short-range magnetic ordering is observed below 5 K. The specific heat measurements down to 1.8 K confirm that no long-range ordering is observed in Ba_3_NbIr_2_O_9_. Electronic structure calculations indicate that the Ir–Ir dimer adopts a homogeneous Ir^3.5+^–Ir^3.5+^. These results highlight a novel design strategy for quantum spin liquid candidates based on targeted manipulation of chemical bonding environments.

## Graphical abstract

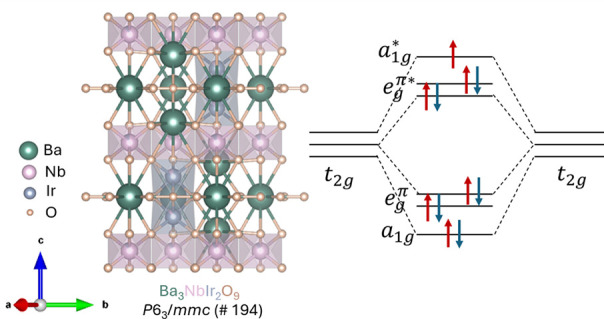

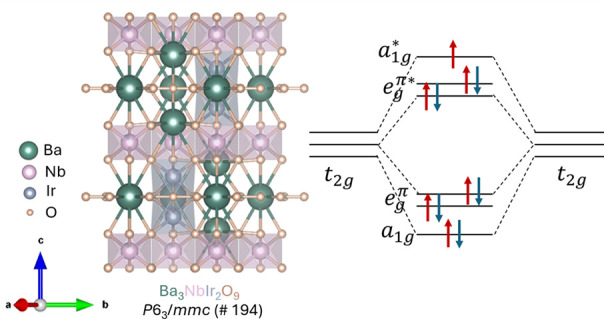

## Introduction

Magnetic frustration arises when competing interactions prevent a system from stabilizing into a well-defined ground state.^1,2^ Traditionally, frustration is associated with specific geometric lattices, such as triangular and pyrochlore structures, where antiferromagnetic (AFM) interactions lead to highly degenerate ground states.^3–6^ However, from a chemical bonding perspective, magnetic frustration can also be viewed as a consequence of electronic instability, where electrons in certain systems fail to reach a stable, well-defined state.^7,8^ Thus, odd-electron bonding theory may be used to predict the emergence of frustration.^9,10^ Odd-electron bonding occurs in molecules and solids where an unpaired electron participates in bonding, leading to fractional bond orders and delocalized magnetic states.^11^ This phenomenon is prevalent in a variety of chemical systems, including organic radicals and diradicals, odd-electron metal–metal bonded clusters, and π-electron delocalized systems.^12,13^ The presence of unpaired electrons in these molecular systems introduces competing magnetic interactions, which can enhance frustration. Specifically, odd-electron systems could exhibit both ferromagnetic (FM) and AFM interactions, leading to degenerate spin configurations and preventing conventional magnetic ordering.^14^ Moreover, the delocalization of odd electrons disrupts long-range magnetic order, promoting the stabilization of exotic quantum phases such as quantum spin liquids (QSLs), where spins remain dynamically correlated rather than forming static magnetic order.^15^

Among various magnetic frustration systems, the Kitaev model on a honeycomb lattice has been a central focus of research in recent years due to its quantum spin-liquid ground state.^16,17^ Particularly, the elementary excitations under an applied magnetic field in Kitaev systems are represented by emergent non-Abelian anyons, which could serve as a key element in topological quantum computation.^18^ The bond-directional magnetic interactions of the Kitaev model can be realized in strongly correlated transition metal compounds, where spin–orbit entangled pseudospins *S* = 1/2 are arranged on a honeycomb lattice of edge-shared octahedra.^19^ Honeycomb-lattice compounds composed of Ir^4+^ or Ru^3+^ ions are prime candidates for the experimental realization of Kitaev spin liquids because their t_2g_^5^ electron configuration supports *S* = 1/2 states with strong spin–orbit coupling (SOC).^20^ Iridium-based oxides with Ir^4+^ in the honeycomb lattice have attracted significant interest due to their unique electronic configurations and strong SOC effects.^21^ Notable examples include Na_4_Ir_3_O_8_ with sole Ir^4+^ as a potential quantum spin-liquid candidate.^22^ However, the presence of mixed-valence Ir ions, coupled with strong SOC, results in complex magnetic interactions that warrant further investigation. In Ba_4_LnIr_3_O_12_, the valence state of Ir plays a crucial role in determining the magnetic ground state: long-range antiferromagnetic order emerges only in compounds with tetravalent Ir (Ln = Ce, Pr, Tb), while others (Ln = La, Nd, Sm–Gd, Dy–Lu) with mixed-valence Ir^+4.33^ remain paramagnetic down to 1.8 K.^23^ In Ba_3_LiIr_2_O_9_, Ba_3_NaIr_2_O_9_, and Ba_3.44_K_1.56_Ir_2_O_10_, the effective magnetic moments extracted from Curie–Weiss fits are low, which is attributed to a combination of strong SOC and antiferromagnetic Ir–Ir exchange interactions.^24^ Notably, Dey et al. established a gapless spin-liquid ground state in Ba_3_InIr_2_O_9_ ^25^ and high-pressure Ba_3_YIr_2_O_9_.^26^ Among these materials, Ba_4_NbIr_3_O_12_ is of particular interest due to its trimer-based structural motif, where IrO_6_ octahedra form face-sharing units.^15^ From the molecular perspective, the Ir trimer contains two Ir^4+^ ions (two unpaired electrons in total) and one Ir^5+^ ion (all paired electrons in Ir^5+^), which creates the 2-electron-3 center (2e^–^–3c) system. The 2-electron-3-center can be considered as a stable system.^27^ Here, we applied high-pressure and high-temperature synthesis to test the idea that we can achieve the odd-electron system in iridates.

In this report, we present the synthesis and characterization of a new iridate compound, Ba_3_NbIr_2_O_9_, obtained via a high-pressure (7 GPa) and high-temperature (1350 °C) solid-state reaction. The material crystallizes in a hexagonal structure with the space group *P*6_3_/*mmc*. Its crystal structure features Ir–Ir dimers with only one unpaired electron per dimer, leading to an electronically unstable one-electron two-center configuration. The formal Ir valence is as low as 3.5+, which is, to our knowledge, the lowest reported among mixed-valence iridates. Magnetic susceptibility measurements reveal a negative Weiss temperature of ∼−27 K, suggesting dominant antiferromagnetic interactions, although no evidence of long-range magnetic order is observed down to 1.8 K. Instead, short-range magnetic correlations develop below approximately 5 K. Electrical resistivity measurements confirm a semiconducting behavior with an estimated band gap of 0.03 eV. A molecular orbital model is proposed to describe the magnetic ground state, supported by density functional theory (DFT) calculations.

## Experimental Section

### High-Pressure High-Temperature Synthesis

Polycrystalline samples of Ba_3_NbIr_2_O_9_ were prepared by a solid-state transformation of a precursor under high-pressure and high-temperature conditions. The ambient-phase precursor, Ba_4_NbIr_3_O_12_, was first prepared by solid-state reaction.^28^ The reagents BaCO_3_, Nb_2_O_5_, and IrO_2_ (Alfa Aesar, powder, 99.9%, 99.5%, and 99.9%, respectively) were thoroughly mixed in stoichiometric ratios and heated in air within alumina crucibles at 900 °C for 25 h. The resulting powders were subsequently reground, pelletized, and reannealed in air at 1100 °C for 48 h. The precursor was packed in a Pt capsule inside an alumina sleeve and loaded into a Walker-type multianvil press^29^ for high-pressure and high-temperature synthesis. The assembly consists of a Ceramacast 646 octahedral pressure medium, a Re heater, and Toshiba–Tungaloy tungsten carbide (WC) anvils.^30^ The sample was then pressurized to 7 GPa at ambient temperature over 24 h. After that, the sample was heated to 1350 °C and maintained at that temperature for 3 h. Then, the sample was quenched to room temperature before depressurizing to ambient pressure. The synthesized Ba_3_NbIr_2_O_9_ sample is metastable, remaining air-stable at ambient conditions. Samples stored for several months in air showed no signs of degradation or phase transformation.

### Crystal Structure Determination

To examine the crystalline structure and detect potential defects in Ba_3_NbIr_2_O_9_ single crystals, a specimen with dimensions of 0.097 × 0.087 × 0.022 mm^3^ was selected for analysis. The crystal was mounted on a nylon loop using Paratone oil. A Rigaku XtaLAB Synergy, Dualflex, Hypix single-crystal X-ray diffractometer was employed for data collection under ambient conditions. Crystallographic data were acquired using the ω scan method, with Mo Kα radiation (λ = 0.71073 Å) emitted from a microfocus sealed X-ray tube, operated at 50 kV and 1 mA. The experimental parameters, including the total number of scans and images, were determined through automated strategy calculations using CrysAlisPro software (version 1.171.42.101a, Rigaku OD, 2023). Subsequent data reduction processes involved corrections for Lorentz and polarization effects. An advanced numerical absorption correction was implemented, leveraging Gaussian integration across a model of a multifaceted crystal.^31^ Additionally, an empirical absorption correction employing spherical harmonics was performed within the SCALE3 ABSPACK scaling algorithm to refine the data further.^32^ The structure was solved and refined using the Bruker SHELXTL Software Package.^33,34^

### Phase Analysis and Chemical Composition Determination

Powder X-ray diffraction (PXRD) analysis was carried out to investigate the sample. The Ba_3_NbIr_2_O_9_ crystals were ground using an agate mortar and pestle to obtain a homogeneous powder. The prepared powder was then evenly spread onto a single-crystalline silicon sample holder designed for zero-background measurements, with minimal application of vacuum grease to secure the powder in place. The PXRD data were collected at room temperature over a 2θ range from 5° to 100°, with an incremental step of 0.01° and a fixed dwell time of 3 s per step. These measurements were performed using a Rigaku MiniFlex II powder diffractometer, employing Bragg– Brentano geometry coupled with Cu Kα radiation (λ = 1.5406 Å). Data refinement was carried out using the GSAS-II software suite.^35^ To analyze the chemical compositions, a JEOL 6610LV scanning electron microscope equipped with a tungsten hairpin emitter was employed. Elemental analysis was conducted via energy-dispersive X-ray spectroscopy (EDS) using an Oxford Instruments AZtec system (Oxford Instruments, High Wycombe, Buckinghamshire, England), operating software version 3.1. The setup included a 20 mm^2^ silicon drift detector (SDD) and an ultrathin window. The single crystals of Ba_3_Ir_2_NbO_9_ were affixed to carbon adhesive tape and introduced into the SEM chamber for examination under an accelerating voltage of 20 kV. Spectra were collected at multiple points along the individual crystals over an optimized time frame (100 s).

### Physical Properties Measurement

Temperature- and field-dependent magnetization were measured with a Quantum Design physical property measurement system (PPMS) under a temperature range of 1.8–300 K, and applied fields up to 9 T. AC susceptibility measurements were also performed at various frequencies (from 10 to 1000 Hz). Electrical resistivity measurements were performed with a four-probe method using platinum wires on a pelletized sample of Ba_3_NbIr_2_O_9_ in the temperature range of 1.8–300 K in the PPMS. The specific heat was measured from 1.8 to 100 K by a PPMS DynaCool equipped with a heat-capacity option.

### Electronic Structure Calculations

Density Functional Theory (DFT) calculations were performed using version 7.3.1 of the Quantum ESPRESSO code to analyze the band structure and density of states (DOS) of Ba_3_NbIr_2_O_9_.^36,37^ The calculations utilized projector augmented-wave (PAW) pseudopotentials in conjunction with the Perdew–Burke–Ernzerhof (PBE) exchange-correlation functional.^38,39^ A wave function cutoff energy of 300 Ry and a charge density cutoff set to 12 times this value were employed. A 9 × 9 × 3 Monkhorst–Pack *k*-points mesh was employed in the reciprocal space.^40^ Convergence tests were conducted to ensure the change of total energy was smaller than 1 meV/atom. The Davidson diagonalization algorithm^41^ was applied, and the convergence threshold for self-consistency was set to 10^–9^ Ry. The high-symmetry path for the Brillouin zone was generated using the Spglib library.^42,43^

## Results and Discussion

The high-pressure phase of Ba_3_NbIr_2_O_9_ crystallizes in the hexagonal *P*6_3_/*mmc* space group, exhibiting higher crystallographic symmetry compared to the rhombohedral *R*-3*m* structure adopted by the ambient-pressure Ba_4_NbIr_3_O_12_. Lattice constants and refined structural parameters obtained from single-crystal X-ray diffraction (SCXRD) are summarized in Tables S1 and S2. As illustrated in Figure 1, Ba_3_NbIr_2_O_9_ contains IrO_6_ octahedra analogous to those in Ba_4_NbIr_3_O_12_; however, only two face-sharing IrO_6_ octahedra are present, forming Ir_2_O_9_ dimers. In contrast, Ba_4_NbIr_3_O_12_ features three face-sharing IrO_6_ units forming Ir_3_O_12_ trimers. In Ba_3_NbIr_2_O_9_, these Ir_2_O_9_ trimers alternate with corner-sharing NbO_6_ octahedra along the *c*-axis, constructing a six-layer (i.e., two repeating units of three MO_6_ octahedra) 6H-type hexagonal perovskite structure. This differs from the ambient-pressure 9R polytype of Ba_4_NbIr_3_O_12_, which comprises a 12-layer stacking sequence.^44^ Consistent with this structural change, the *c*-axis lattice parameter of the high-pressure phase is approximately halved relative to that of the ambient phase. In addition to the distinct Ir–Ir bonding motifs—dimers in Ba_3_NbIr_2_O_9_ and trimers in Ba_4_NbIr_3_O_12_—the geometries of the Ir_2_O_9_ and Ir_3_O_12_ structural units differ significantly between the two Ba–Nb–Ir–O phases, as depicted in Figure 2. In Ba_4_NbIr_3_O_12_, the Ir_3_O_12_ trimers form a regular triangular lattice viewed along the *c*-axis, whereas in Ba_3_NbIr_2_O_9_, the Ir_2_O_9_ dimers adopt a regular honeycomb arrangement. Similar structural trends are observed in the Bi analogs, Ba_4_BiIr_3_O_12_ and Ba_3_BiIr_2_O_9_, both of which crystallize in orthorhombic structure types.^45,46^ In these compounds, the Ir_3_O_12_ and Ir_2_O_9_ units form distorted triangular and distorted honeycomb lattices, respectively. Cation ordering also differs between the two structures: Ba_4_NbIr_3_O_12_ exhibits complete Nb:Ir sublattice ordering, whereas in Ba_3_NbIr_2_O_9_, partial substitution of Nb by Ir and vice versa is observed, with a refined occupancy ratio of approximately 9:1.

**Figure 1:**
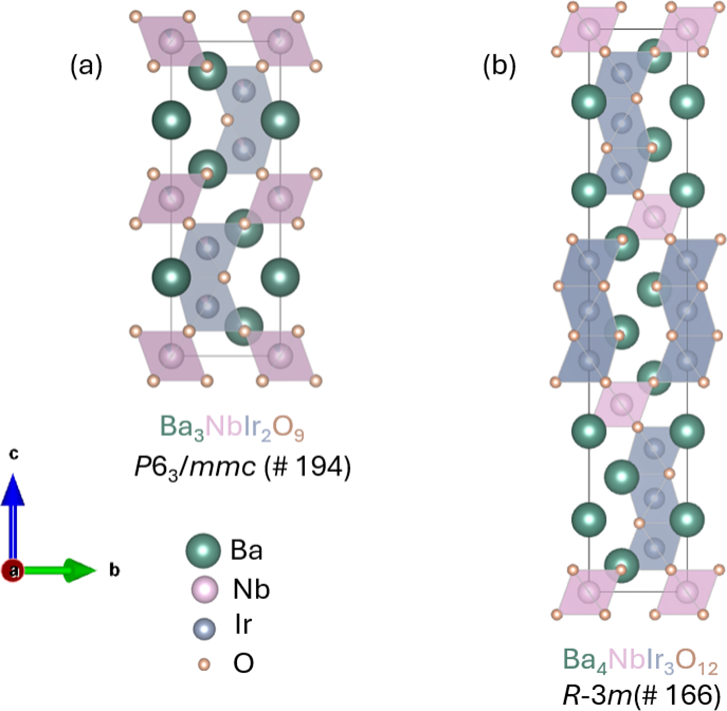
(a) Crystal structures of Ba_3_NbIr_2_O_9_ obtained under high pressure and high temperature; (b) Crystal structure of ambient-pressure Ba_4_NbIr_3_O_12_.

**Figure 2:**
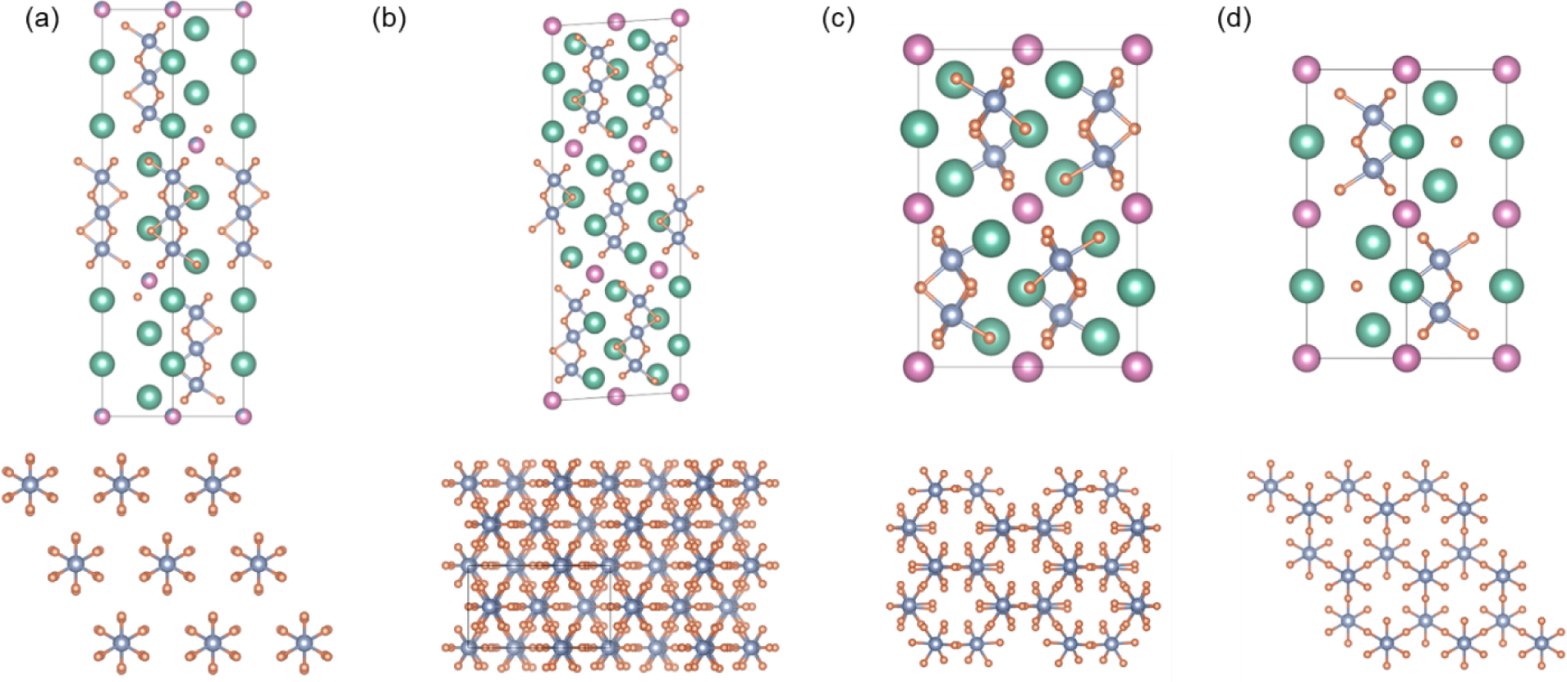
Crystal structures of (a) Ba_4_NbIr_3_O_12_, (b) Ba_4_BiIr_3_O_12_, (c) Ba_3_BiIr_2_O_9_, and (d) Ba_3_NbIr_2_O_9_ highlighting the arrangement of Ir–O polyhedra within the layered frameworks. The figures emphasize the distinct geometrical patterns formed by Ir_3_O_12_ trimers and Ir_2_O_9_ dimers, which assemble into triangular and honeycomb lattices, respectively.

PXRD analysis (Figure 3), based on a calculated pattern from the SCXRD-derived CIF file, confirms the phase identity and high yield (>90%) of Ba_3_NbIr_2_O_9_ synthesized from the precursor Ba_4_NbIr_3_O_12_. A minor impurity phase of IrO_2_ (2.6 wt %) is detected, but given its nonmagnetic nature and low concentration, it is not expected to significantly affect the physical property measurements. Rietveld refinement yields a final weighted R-factor (wR) of 9.12% based on 8,501 observed intensity data points and a conventional R-factor (R) of 5.78%, which is considered satisfactory given the limited sample quantity and internal stress commonly associated with high-pressure-synthesized metastable phases. The refined structural model is consistent with our SCXRD data, validating the assigned space group and atomic positions. Scanning electron microscopy with energy-dispersive X-ray spectroscopy (SEM-EDS) was further employed to verify the phase composition and homogeneity (Figure S1). Multiple measurements across the sample yielded a consistent average atomic ratio of O:Ba: Ir:Nb = 59(1):21(1):13(1):7(1), indicating good agreement in chemical composition with X-ray measurements.

**Figure 3:**
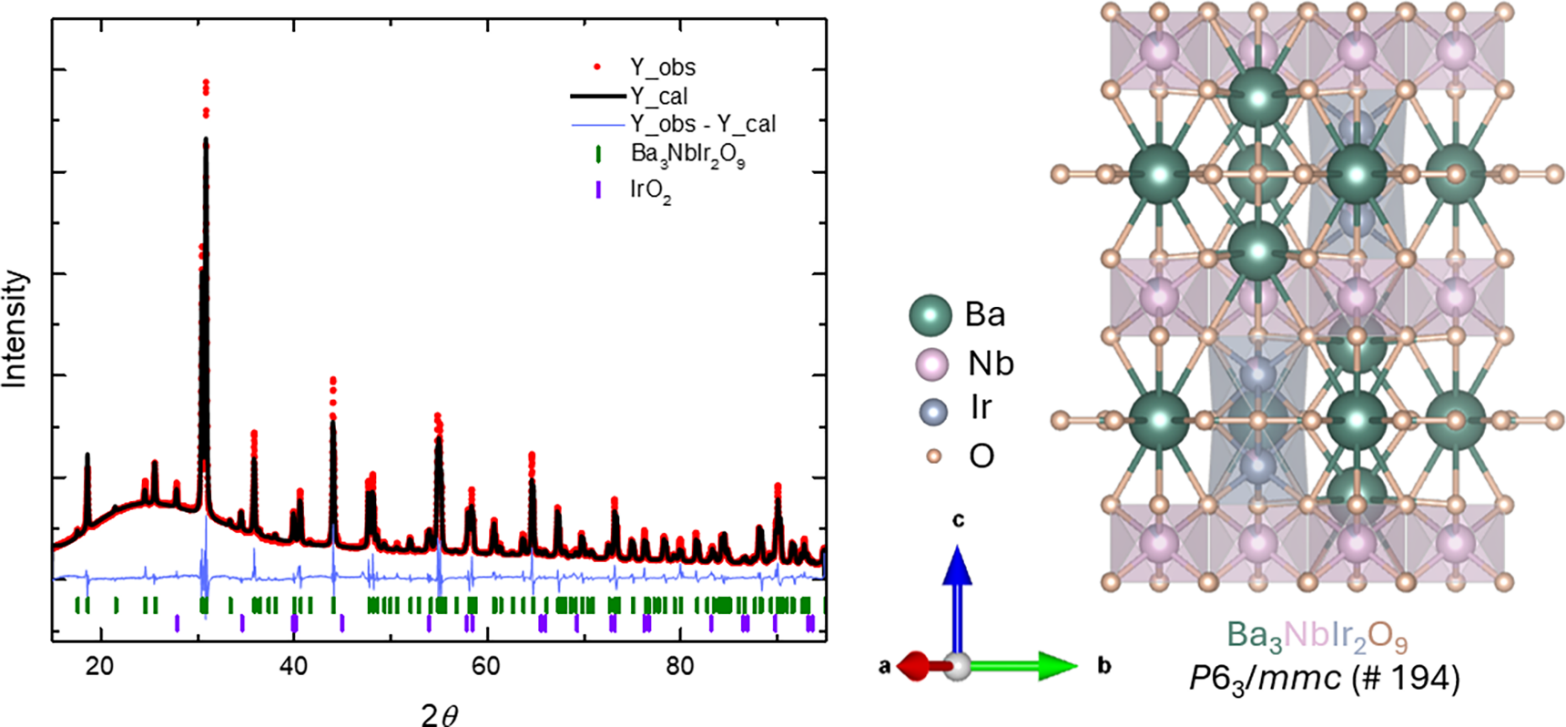
Powder X-ray diffraction pattern of the high-pressure Ba_3_NbIr_2_O_9_ phase. Experimental data (red circles) were fitted using Rietveld refinement (black line). The difference curve (blue line) represents the residual between observed and calculated patterns. Bragg reflection positions for Ba_3_NbIr_2_O_9_ and IrO_2_ are indicated by green and purple tick marks, respectively.

To investigate the magnetic behavior of the high-pressure phase, temperature-dependent magnetization measurements were performed using Zero-Field-Cooled Warming (ZFCW) and Field-Cooled (FC) protocols, as shown in Figure 4a. No significant difference between the ZFCW and FC curves is observed between 5 and 300 K under magnetic fields ranging from 1 kOe to 90 kOe. However, the upper inset of Figure 4a reveals a bifurcation between ZFCW and FC curves below 5 K at 1 kOe and 10 kOe, indicating the emergence of a frozen spin state. The lower inset shows the temperature derivative of the magnetization normalized by field, d­(*MT*/*H*)/d*T*, where *M* is magnetization, *T* is temperature, and *H* is magnetic field. Anomalies in this derivative at low temperatures further support the presence of a magnetic transition associated with spin freezing. In Figure 4b, magnetic susceptibility data at 1 kOe have been subtracted from those at higher fields to eliminate the contribution of a diamagnetic background signal—likely arising from impurities. At temperatures above 25 K, the susceptibility curves at all fields >1 kOe converge, indicating intrinsic paramagnetic behavior. Therefore, a representative Curie–Weiss fit was performed on the 30 kOe data. The high-temperature susceptibility data (25–300 K) at 30 kOe are well described by the modified Curie–Weiss law (eq 1), as shown in Figure 4c:

1 $$\chi =\chi_{0}+\frac{C}{T-\theta_{\mathrm{cw}}}$$

where χ_0_ is the temperature-independent susceptibility, *C* is the Curie constant, and θ_cw_ is the paramagnetic Curie temperature. The least-squares fitting yields χ_0_ = 1.08 × 10^–4^ emu mol^–1^Oe^–1^ and θ_cw_ = −26.9(1) K, which is indicative of antiferromagnetic interdimer exchange. The effective magnetic moment μ_eff_ = 1.01(1) μ_B_/f.u., which is smaller than Hund’s-rule value of 1.73 μ_B_/f.u. for *S* = 1/2, while the reported μ_eff_ = 0.8 μ_B_/f.u. for ambient-pressure Ba_4_NbIr_3_O_12_. While the frustration index *f* = |θ_cw_|/*T*_M_ = 5.4 does not meet the conventional threshold for a highly frustrated magnet, we note that a modest *f* value does not necessarily preclude the possibility of a spin liquid ground state, especially in systems with significant quantum fluctuations or geometrical frustration.^47^ Figure 4d displays the field-dependent magnetization of high-pressure Ba_3_NbIr_2_O_9_ up to 9 T at various temperatures. At 1.8 K, the curve exhibits a clear hysteresis loop without reaching saturation, suggesting the presence of short-range ferromagnetic correlations. At temperatures above 10 K, a linear field dependence is observed, consistent with a transition to a paramagnetic state described by Curie–Weiss behavior.

**Figure 4:**
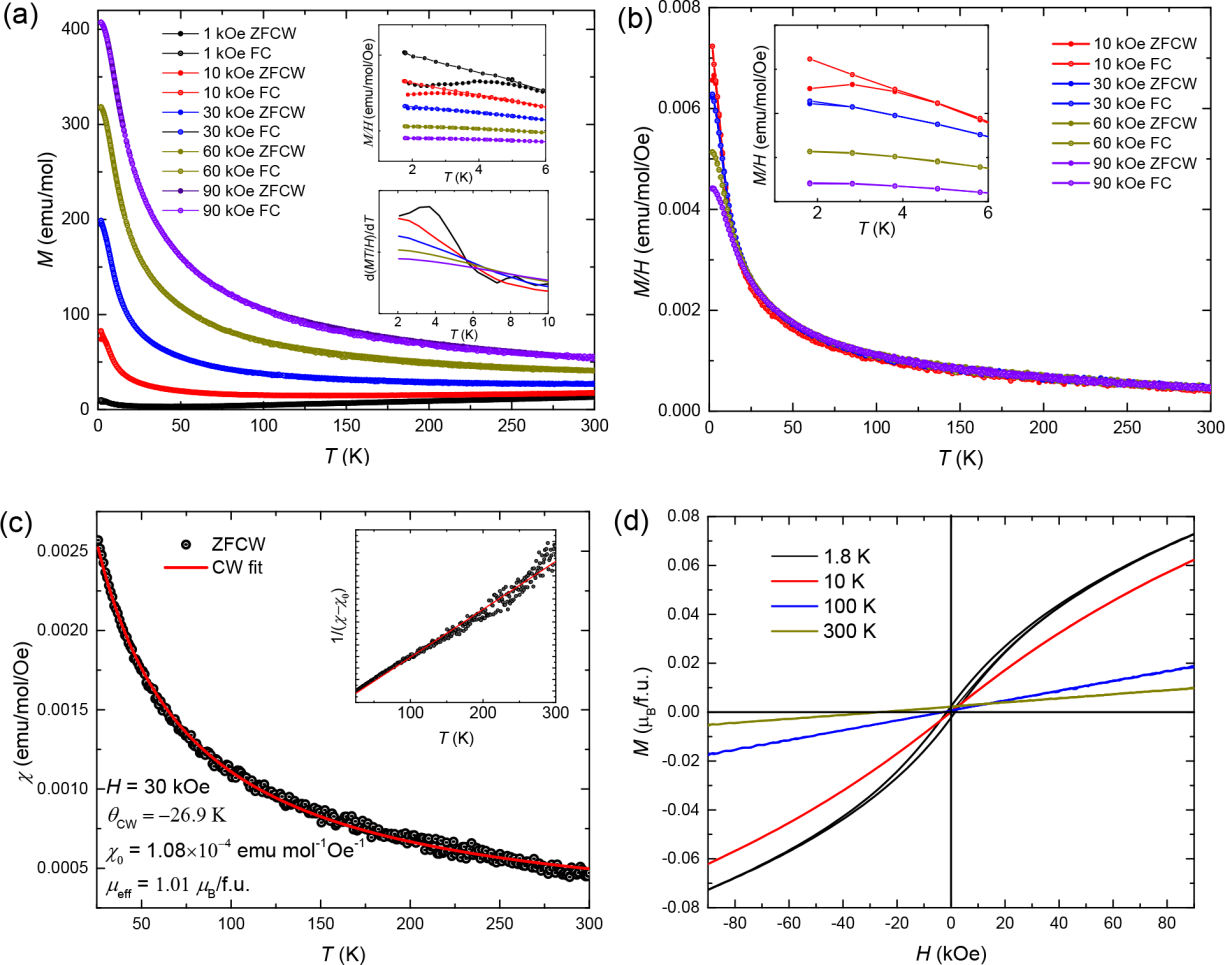
(a) Temperature-dependent magnetization measured at various magnetic fields with Zero-Field-Cool-Warming (ZFCW) and Field-Cool (FC) temperature protocols. The upper inset highlights magnetic susceptibility in the low-temperature region. The lower inset displays the temperature derivative of the value d­(*MT*/*H*)/d*T*. (b) Temperature-dependent magnetic susceptibility measured under different magnetic fields, with the 1 kOe data subtracted as a reference. (c) Curie–Weiss fitting of the magnetic susceptibility data collected in the ZFCW mode under an applied field of 30 kOe, over the temperature range 25–300 K. The inset shows the linear fitting of 1/(χ – χ_0_) as a function of temperature. (d) Isothermal magnetization curves as a function of magnetic field up to 9 T at selected temperatures.

The temperature-dependent specific heat of high-pressure Ba_3_NbIr_2_O_9_ measured under zero applied magnetic field over the range of 1.8–100 K is shown in Figure 5a. For comparison, specific heat data collected under a 9 T field (Figure S2) show no appreciable deviation, indicating negligible field dependence. No λ-type anomaly is observed, suggesting the absence of long-range magnetic ordering within this temperature window.

**Figure 5:**
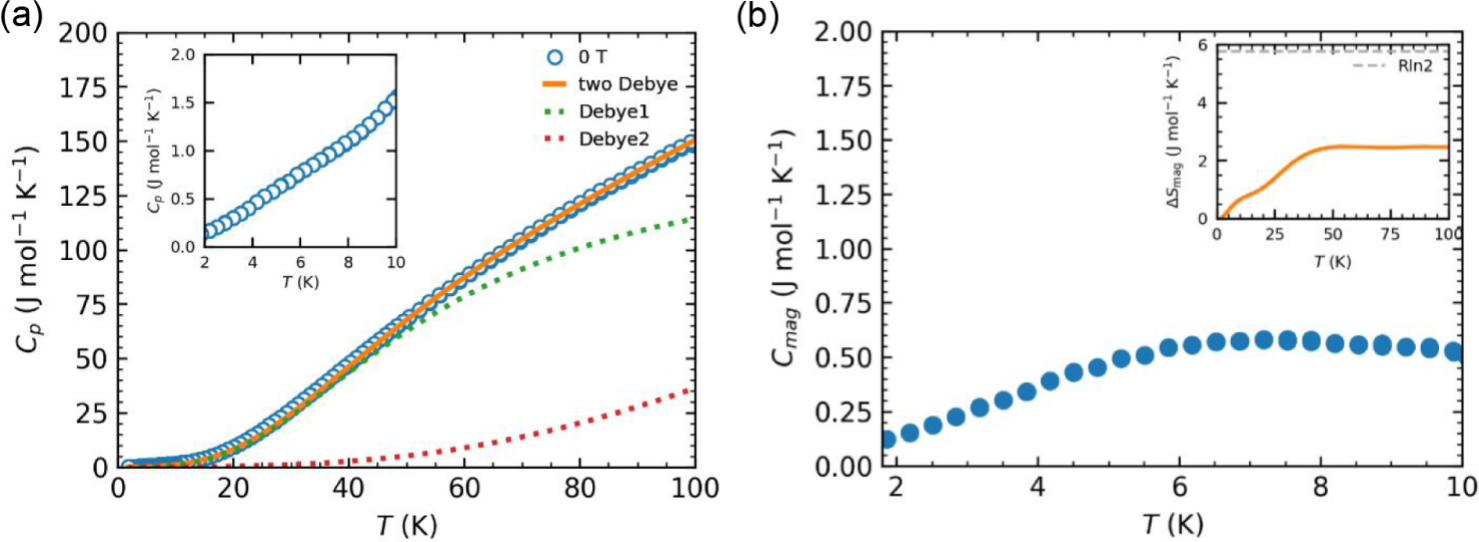
(a) Temperature dependence of specific heat per mole formula unit for high-pressure Ba_3_Nb_2_IrO_9_ measured under zero magnetic field. The solid line represents the phonon contribution (*C*_phonon_), modeled using a two-Debye model. (b) Magnetic contribution to specific heat (*C*_mag_) in the low-temperature range of 1.8–10 K, obtained by subtracting *C*_phonon_ from the total specific heat. The inset shows the corresponding magnetic entropy change (Δ*S*_mag_) as a function of temperature.

To investigate the low-temperature behavior of the specific heat, we plotted *C*_p_/*T* versus *T* ^2^, as shown in Figure S3. In a conventional nonmagnetic insulator, where the heat capacity is dominated by acoustic phonons, this plot should yield a constant at low temperatures. However, our data show a clear deviation from this expected Debye behavior. This behavior rules out conventional phonon dominance in this regime and supports the presence of low-energy spin excitations, consistent with our interpretation of the system as being in a quantum-disordered or spin-liquid-like state.

At higher temperatures, the specific heat is dominated by phonon contributions (*C*_phonon_). Due to the lack of a suitable nonmagnetic analog, the phonon contribution was modeled using a functional form consistent with a harmonic phonon system, obtained by least-squares fitting of the high-temperature (50–100 K) data.^15^ Neither the Debye model (eq 2) nor the Einstein model (eq 3) alone adequately describes the data (see Figure S4):

2 $$C_{D}=9nR{\left(\frac{T}{\theta_{D}}\right)}^{3}\int_{0}^{\theta_{D}/T}\frac{x^{4}e^{x}}{{\left(e^{x}-1\right)}^{2}}dx$$

3 $$C_{E}=3nR{\left(\frac{\theta_{E}}{T}\right)}^{2}e^{\theta_{E}/T}{\left(e^{\theta_{E}/T}-1\right)}^{-2}$$

where *n* is the number of atoms per formula unit, *R* is the gas constant, θ_D_ is the Debye temperature, and θ_E_ is the Einstein temperature.

As shown in Figure 5a, the lattice contribution is well reproduced by the two-Debye model (eq 4), in which the total number of atoms per formula unit, *n*, is fixed, and the oscillator strength *s*_D1_ = 5.8(1) defines the distribution between two Debye modes, with fitted Debye temperatures: θ_D1_ = 226(2) K, θ_D2_ = 757(9) K.

4 $$C=9s_{D1}R{\left(\frac{T}{\theta_{D1}}\right)}^{3}\int_{0}^{\theta_{D1}/T}\frac{x^{4}e^{x}}{{\left(e^{x}-1\right)}^{2}}dx+9\left(n-s_{D1}\right)R{\left(\frac{T}{\theta_{D2}}\right)}^{3}\int_{0}^{\theta_{D2}/T}\frac{x^{4}e^{x}}{{\left(e^{x}-1\right)}^{2}}dx$$

This model is constructed to ensure that the high-temperature limit recovers the expected Dulong–Petit value of 3*nR*, providing a physically constrained and nonarbitrary fit to the phonon background. The magnetic contribution to the specific heat, *C*_mag_(*T*) = *C*(*T*) – *C*_phonon_(*T*), is shown in Figure 5b. A broad hump is observed, corresponding to spin freezing and coinciding with the bifurcation in ZFC/FC magnetization data. For a system with the minimal spin–orbital degeneracy of 2, the associated change of magnetic entropy is expected to approach $\Delta S_{\mathrm{mag}}\left(T\right)=\int \left(C_{\mathrm{mag}}/T\right)dT=R\ln2$ . However, the experimentally estimated effective magnetic moment is smaller than that of a spin-1/2 system, resulting in a suppressed magnetic entropy change. This reduction likely reflects strong SOC effects, which can lower the effective moment and magnetic entropy change via spin–orbital interactions. Similar reductions in the magnetic entropy change have also been reported in other iridate systems such as Ba_3_ScIr_2_O_9_ and Ba_3_YIr_2_O_9_.^48^

It is tempting to attribute the ZFC/FC divergence seen in the DC susceptibility and the linear *C*_mag_ vs *T* data at low temperatures to spin-glass physics. However, we have measured the AC susceptibility of the high-pressure Ba_3_NbIr_2_O_9_ sample at several different frequencies (ranging from 10 to 1000 Hz) and found no evidence of spin glassiness. As shown in Figure 6, the real part of the ac susceptibility, χ′(*T*), shows no discernible frequency dependency. A conventional spin glass will show a maximum in χ′ at the freezing temperature *T*_f_, which then depends strongly on the frequency of measurement.^49^ Therefore, we believe the observed ZFC/FC splitting alone is not sufficient to assign a spin-glass ground state. As noted by Yoshihiko Okamoto et al.,^22^ the slight bifurcations do not represent a contribution from the majority of spins and may originate from a small fraction of impurities, since the difference between ZFC and FC magnetizations is less than 10% of the total signal—which is also the case in our sample. This glassy feature vanishes under applied magnetic fields above 3 T, suggesting it is not intrinsic to the bulk. In contrast, at high field, susceptibility tends to saturate and approach a finite value as *T* → 0, indicating that the majority of the system remains a paramagnetic liquid at least down to 2 K. Also, the specific heat data is field-independent as shown in Figure S2. This is contrary to typical spin-glass behavior. The broad hump observed in the magnetic heat capacities may suggest a crossover into a QSL state, consistent with observations in known QSL candidates such as κ-(BEDT-TTF)_2_Cu_2_(CN)_3_.^50^

**Figure 6:**
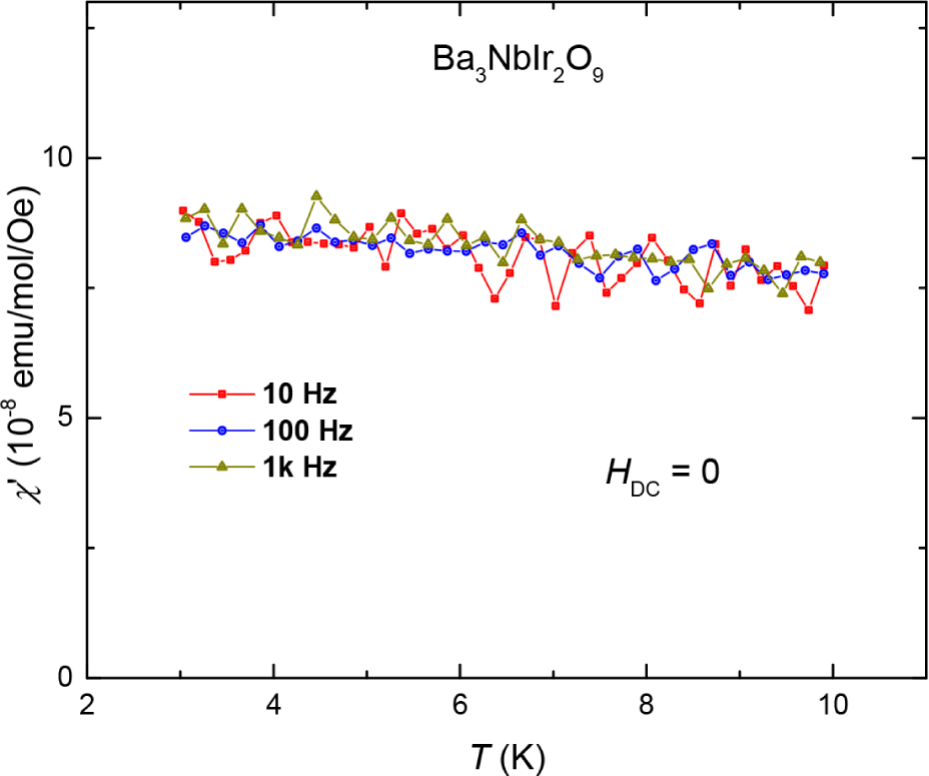
Temperature-dependent AC magnetic susceptibility measured at various frequencies.

The temperature dependence of the dimensionless resistance of high-pressure Ba_3_NbIr_2_O_9_ under magnetic fields up to 9 T is shown in Figure 7a, covering the range from 10 to 300 K. The resistance decreases with increasing magnetic field up to 1 T, a behavior attributed to the gradual alignment of magnetic moments, consistent with the system not reaching full magnetic saturation even under 9 T (see discussion above). The system exhibits overall nonmetallic resistivity, but no single transport model can describe the full temperature range. In the temperature range of 50–200 K in Figure S5, the resistance follows an Efros–Shklovskii variable-range hopping (ES-VRH)^51^ behavior by the form (eq 5), suggesting strong localization and Coulomb interactions in a disordered insulating state.^52^

5 $$R\left(T\right)\propto e^{[{\left(\frac{T_{0}}{T}\right)}^{1/2}]}$$

**Figure 7:**
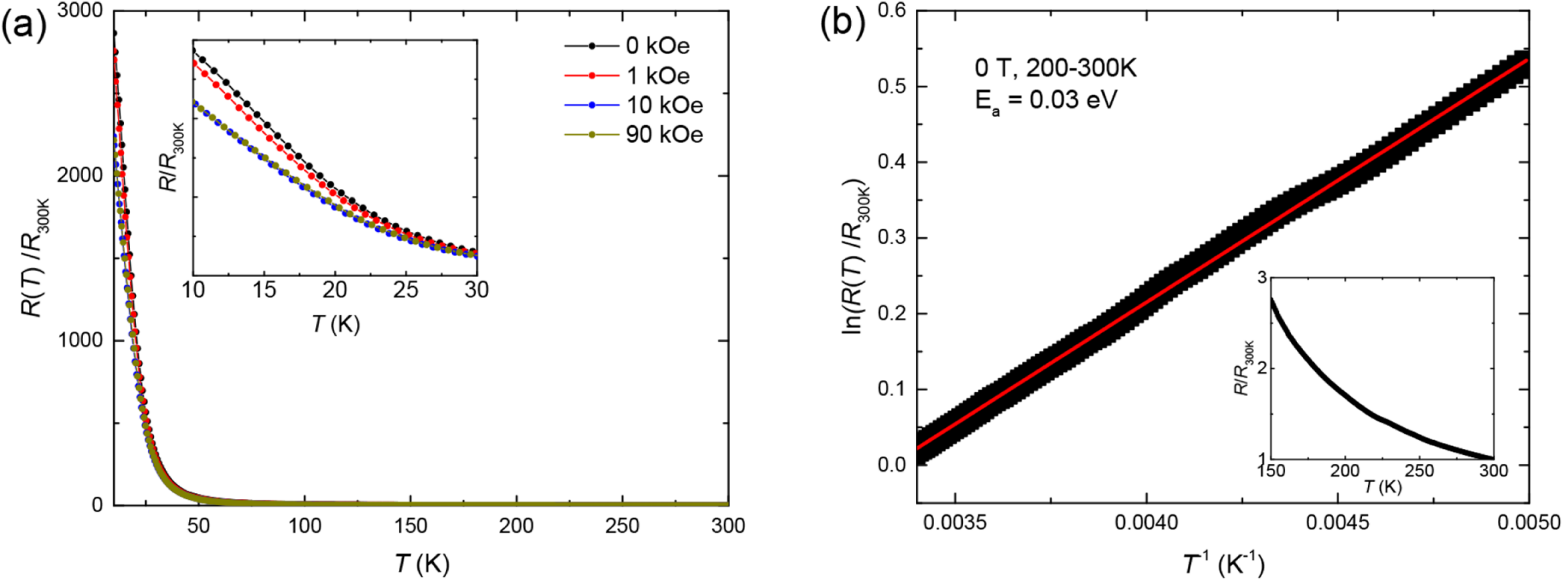
(a) Temperature dependence of dimensionless resistance (divided by the resistance at 300 K for each applied field). The inset emphasizes the rapid upturn in resistance below ∼30 K. (b) Dimensionless resistance ranging from 200 to 300 K was fitted by Arrhenius law. A linear relationship is obtained.

The high-temperature regime (200–300 K) follows an Arrhenius-type thermal activation behavior, $R\left(T\right)\propto e^{E_{a}/k_{B}T}$ , with an activation energy of *E*_a_ = 0.03 eV, as shown in Figure 7b. This confirms that Ba_3_NbIr_2_O_9_ synthesized under high pressure behaves as a semiconductor with thermally activated conduction at high temperatures.

At low temperatures (below ∼50 K), the resistance increases steeply with decreasing temperature, suggesting a transition to a more localized electronic state. However, the data cannot be adequately described by a single thermal activation or variable-range hopping model. This deviation may signal the suppression of charge carrier mobility due to the freezing of electronic hopping processes, the emergence of short-range magnetic ordering, or the onset of spin-glass-like behavior.

The magnetic ground state of Ba_3_NbIr_2_O_9_ is of significant interest, particularly due to the presence of mixed-valent Ir ions. As both Ba^2+^ and Nb^5+^ are nonmagnetic, the magnetic properties of this compound arise primarily from the intra- and interdimer interactions between Ir ions within the Ir_2_O_9_ units. Previous work by Terzić et al. on Ba_5_AlIr_2_O_11_ suggested the presence of charge ordering between Ir^4+^ and Ir^5+^ ions within Ir–Ir dimers.^53^ This was supported by the observation of two inequivalent Ir–O bond distances and anomalies in resistivity, dielectric constant, and specific heat near a characteristic temperature *T*_s_ = 210 K, indicating enhanced charge ordering. In contrast, our structural analysis of Ba_3_NbIr_2_O_9_ reveals identical Ir–O bond lengths and the absence of anomalies in resistivity and specific heat, suggesting no long-range charge ordering in this system.

In 5d^5^ systems such as Sr_2_IrO_4_, strong spin–orbit coupling (SOC) splits the t_2g_ manifold into a fully filled *J*_eff_ = 3/2 band and a half-filled *J*_eff_ = 1/2 band, with even a moderate on-site Coulomb repulsion *U* sufficient to open a Mott gap. However, Ba_3_NbIr_2_O_9_ represents a 5d^5.5,^ system, where the *J*_eff_ = 1/2 band becomes more than half-filled, leading to partial occupation of the upper Hubbard band. This electronic configuration is fundamentally distinct from that of the 5d^5^ system in Sr_2_IrO_4_, where a half-filled *J*_eff_ = 1/2 band enables a SOC-assisted Mott insulating state. To explore the electronic structure of Ba_3_NbIr_2_O_9_, we first carried out nonmagnetic GGA calculations. As shown in Figure 8a, the t_2g_ manifold, which accommodates the Fermi level, is well separated from the higher-energy e_g_ states by an energy gap of approximately 2 eV. The projected density of states (PDOS) in Figure 8b reveals strong hybridization between the Ir 5d and O 2p orbitals in the energy range from −2.1 eV up to the Fermi level. In contrast, the Ba 5p and O 2s states are located deeper in energy, near −12 eV and −19 eV, respectively, while unoccupied Nb 4d states appear just above the Fermi level in the 2.2–2.5 eV range, supporting a Nb^5+^ (4d^0^) configuration. These results highlight the covalent nature of Ir–O bonding and suggest a complex interplay between crystal field effects, covalency, and SOC in shaping the low-energy electronic structure.

**Figure 8:**
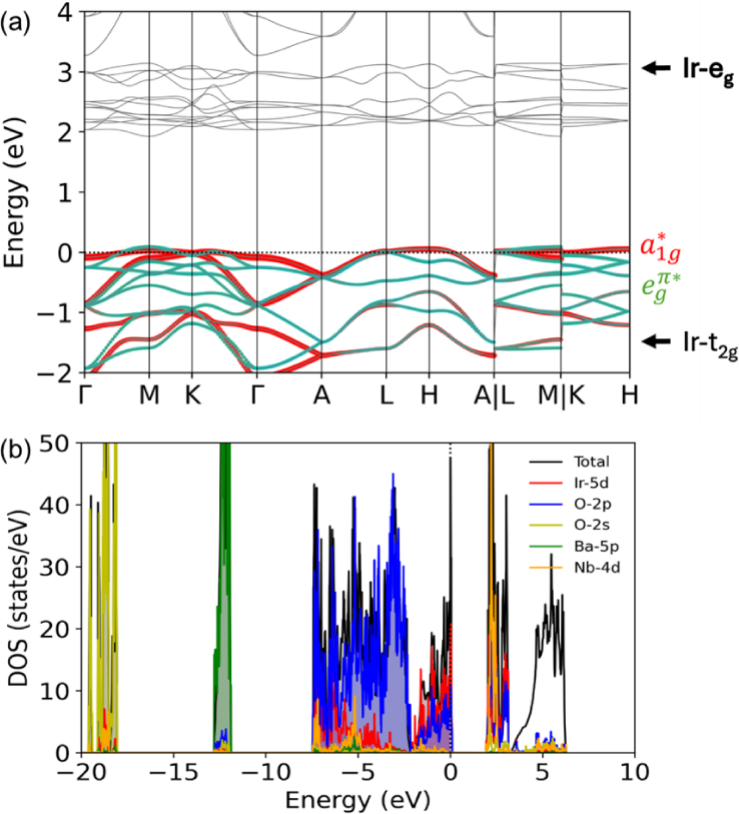
Electronic structure of Ba_3_NbIr_2_O_9_ obtained from nonmagnetic GGA calculations: (a) band structure showing that the t_2g_ manifold, which hosts the Fermi level, is well separated from the higher-energy e_g_ states by approximately 2 eV; (b) projected density of states (PDOS), revealing strong hybridization between Ir 5d and O 2p orbitals near the Fermi level, while Ba 5p and O 2s states reside at deeper energies (∼−12 eV and ∼−19 eV, respectively). Nb 4d states appear above the Fermi level in the range of 2.2–2.5 eV.

Applying a Hubbard *U* = 4 eV does not induce an insulating gap in Ba_3_NbIr_2_O_9_, but it increases the energy separation between the partially occupied bands near the Fermi level, as shown in Figure 9a. This behavior highlights the delocalized nature of the antibonding molecular orbitals, consistent with their broad bandwidth. When spin–orbit coupling (SOC) is included, the band structure (Figure 9b) shows that these two bands become fully split along the high-symmetric path Γ-M-K-Γ-A, evidencing the removal of band degeneracy due to SOC. These results underscore the importance of both electron correlations and SOC in shaping the low-energy electronic structure, although neither alone is sufficient to open a band gap (Figure 9c).

**Figure 9:**
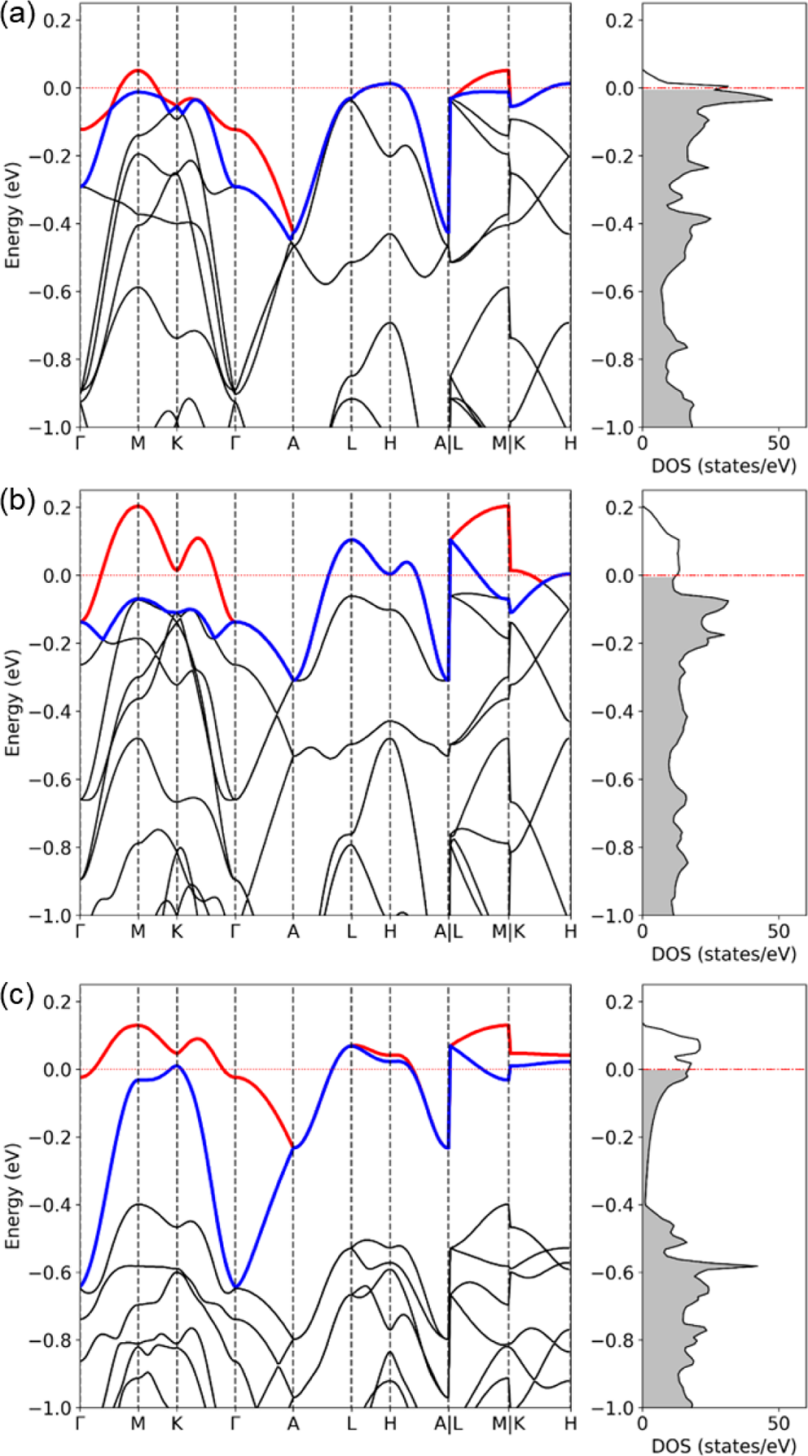
Calculated band structures of Ba_3_NbIr_2_O_9_ under different levels of theoretical treatment: (a) GGA + *U* (with *U* = 4 eV) increases band separation but fails to open a gap, indicating the delocalized character of these orbitals; (b) GGA + SOC lifts the band degeneracy along the high-symmetry path Γ-M-K-Γ-A; (c) GGA + SOC + *U* fails to open the band gap.

A more appropriate framework may be the molecular orbital picture, previously introduced by Sergey V. Streltsov for transition metal compounds^54^ and applied to 6H-type dimerized systems such as Ba_3_MRu_2_O_9_ (M = In, Y, Lu)^55^ and Ba_3_InIr_2_O_9_,^56^ similarly applicable to our Ir–Ir dimers. Accordingly, Ir ions in a strong octahedral crystal field environment give rise to well-separated t_2g_ and e_g_ manifolds shown in Figure 10. A trigonal distortion (characteristic of the *P*6_3_/*mmc* structure) further splits the t_2g_ levels into an a_1g_ singlet and an e_g_^π^ doublet. The resulting antibonding molecular orbitals e_g_^π^∗ and a_1g_∗ hybridize and dominate the electronic states near the Fermi level. The insulating behavior of Ba_3_NbIr_2_O_9_ originates from a cooperative interplay of Hund’s coupling, crystal field effects, SOC, and strong intradimer hopping. With one minority-spin electron per dimer occupying the antibonding a_1g_∗ orbital, the system is sensitive to magnetic ordering. Our calculations (details to be reported elsewhere) explored four magnetic configurations: ferromagnetic (FM), fully antiferromagnetic (AFM1), ferromagnetic intradimer and antiferromagnetic interdimer (AFM2), and antiferromagnetic intradimer with ferromagnetic interdimer (AFM3). Only the AFM2 configuration yields an insulating ground state with the lowest total energy. In this state, each Ir–Ir dimer maintains a ferromagnetic alignment, allowing bonding–antibonding orbital formation, while the antiferromagnetic arrangement between dimers suppresses interdimer hopping and prevents band overlap, thereby opening a band gap. A similar mechanism has been proposed in related compounds such as Ba_3_LiIr_2_O_9_^58^, Ba_3_YIr_2_O_9_^59^, and Ba_3_InIr_2_O_9_^60^, reinforcing the idea that insulating behavior in these iridate dimers is a result of both orbital hybridization and specific magnetic ordering.

**Figure 10:**
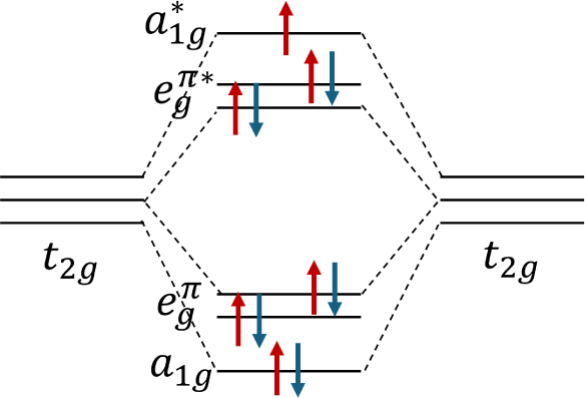
Energy-level and spin occupation diagram for a hybridized Ir^3.5+^–Ir^3.5+^ dimer.

Despite significant interest in (5d^3.5^),^24,57^ (5d^4^),^60,61–67^ (5d^4.5^),^60,26,48,58,25,56,59^ and (5d^5^)^60,68–70^ iridates, systems containing Ir with lower oxidation states have remained largely unexplored. Figure 11 presents the dependence of the Ir–Ir interatomic distance (*d*(Ir–Ir)) and the average Ir–O bond length (*d*(Ir–O)) on the average Ir valence (ν­(Ir)). This work represents the first systematic investigation extending to Ir valence states as low as +3.5 (i.e., 5d^5.5^). Overall, *d*(Ir–Ir) increases with rising ν­(Ir), consistent with enhanced electrostatic repulsion between Ir cations at higher oxidation states. Similar behavior has been reported in the structurally analogous Ba_3_MRu_2_O_9_ ruthenium systems. Notable deviations from this trend are observed in Ba_3_Ti^4+^Ir_2_^4+^O_9_ and Ba_3_Nb^5+^Ir_2_^3.5+^O_9_, likely due to cation disorder affecting local bonding environments. In contrast, the average *d*(Ir–O) decreases with increasing ν­(Ir), which is attributed to the stronger Coulombic attraction between more highly charged Ir cations and surrounding oxygen anions, despite the concurrent increase in Ir–Ir repulsion.

**Figure 11:**
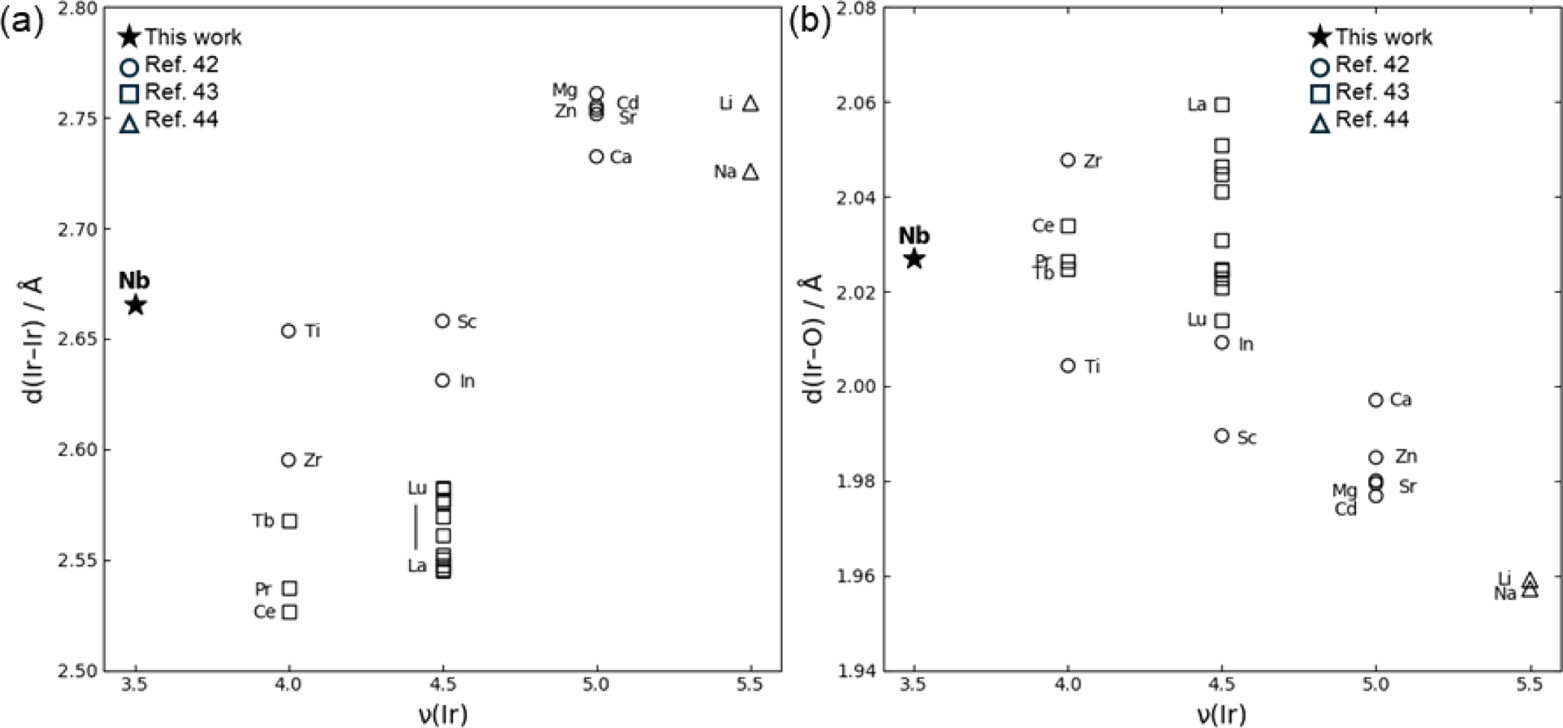
(a) Variation of the Ir–Ir interatomic distance, *d*(Ir–Ir), as a function of the average Ir valence, ν­(Ir). (b) Variation of the average Ir–O bond length, *d*(Ir–O), as a function of ν­(Ir). Comparative data from other Ba_3_MIr_2_O_9_ compounds are included: M = Ti, Zr^60^ and Ce, Pr, Tb^61^ for Ir^4+^; M = Sc, In^60^ and various lanthanides^61^ for Ir^4.5+^; M = Mg, Ca, Sr, Zn, Cd^60^ for Ir^5+^; and M = Li, Na^24^ for Ir^5.5+^.

## Conclusion

In summary, we reported a new Ba_3_NbIr_2_O_9_ phase obtained under high-pressure and high-temperature conditions. Ba_3_NbIr_2_O_9_ crystallizes in the 6-layer 6H hexagonal perovskite unit cell with the *P*6_3_/*mmc* space group. The negative Weiss temperature of −26.9 K and short-range magnetic ordering near 5 K indicate that it is a geometrically moderately frustrating magnet. No anomalies suggesting long-range magnetic ordering were observed in the specific heat measurements, while the hump aligning with the bifurcation point in the ZFC/FC susceptibility indicates the buildup of short-range magnetic ordering. Resistivity measurements with a gap of 0.03 eV indicate that it is a semiconductor. Such a system may offer a promising platform to unravel the interplay between SOC, frustration, and electronic correlations.

## Supplementary Material



## Notes

Single crystal X-ray diffraction crystallographic details of Ba_3_NbIr_2_O_9_; EDS measurement results; temperature-dependent on specific heat under 0 and 9 T; low-temperature heat capacity plotted as *C*_p_/*T* vs *T* ^2^; specific heat data fitted by Debye and Einstein model; resistance fitted at low temperature range (PDF)
