# Supplementary material for: Single-Electron Bond in Ir–Ir Dimer Stabilized under Pressure
Source: Inorg Chem. 2025 Nov 11;64(46):23097–107. doi: 10.1021/acs.inorgchem.5c04558 (PMC12648646; doi:10.1021/acs.inorgchem.5c04558)

Supplementary Information

Single-electron Bond in Ir-Ir Dimer Stabilized under Pressure

Cheng Peng[1], Mingyu Xu[1], Jie Li[2], Weiwei Xie[1*]

1. Department of Chemistry, Michigan State University, East Lansing, MI, 48824 USA
2. Department of Earth and Environmental Sciences, University of Michigan, Ann Arbor, MI, 48109 USA

Corresponding Author: Weiwei Xie (xieweiwe@msu.edu)

Table of Contents

Table S1. The crystal structure and refinement of $Ba_3NbIr_2O_9$……………………………….……....S2

Table S2. Atomic coordinates and equivalent isotropic atomic displacement parameters…..S2

Fig. S1. EDS measurement results……………………………………………………………….S3

Fig. S2. Temperature-dependent of specific heat under 0 T and 9 T ……………………….……..S4

Fig. S3. Low-temperature heat capacity plotted as Cp/T vs. T^2…………...……………….……..S4

Fig. S4. Specific heat data fitted by Debye and Einstein model ………………………………….S5

Fig. S5. Resistance fitted at low temperature range ……………………………………………..S5

Table S1. The crystal structure and refinement of $Ba_3Ir_2NbO_9$ at room temperature 300 K (Mo Kα radiation). Values in parentheses are estimated standard deviation from refinement.

**Chemical Formula**	$\mathbf{Ba_3Ir_2NbO_9}$
Formula Weight	1045.74 g/mol
Space Group	$P6_3/mmc$
Unit Cell dimensions	a = 5.75897(11) Å<br>b = 5.75897(11) Å<br>c = 14.3577(3) Å
Volume	412.386(18) $Å^3$
Z	2
Density (calculated)	8.202 g/cm^3
Absorption coefficient	45.403 mm^{-1}
F (000)	859.0
2θ range	8.172 to 82.286°
Reflections collected	24081
Independent reflections	573 [R_{int} = 0.0904]
Refinement method	Full-matrix least-squares on F^2
Data/restraints/parameters	573/0/24
Final R indices	R_1 (I>2σ(I)) = 0.0184; wR_2 (I > 2 σ(I)) = 0.0400<br>R_1 (all) = 0.0206; wR_2 (all) = 0.0405
Largest diff. peak and hole	+1.72 $e^-/Å^3$ and -1.58 $e^-/Å^3$
R. M. S. deviation from mean	0.252 $e^-/Å^3$
Goodness-of-fit on F^2	1.376

Table S2. Atomic coordinates and equivalent isotropic atomic displacement parameters ($Å^2$) of $Ba_3NbIr_2O_9$. (U_{eq} is defined as one-third of the trace of the orthogonalized U_{ij} tensor.)

$\mathbf{Ba_3NbIr_2O_9}$	**Wyck.**	***x***	***y***	***z***	**Occ.**	$\mathbf{U_{eq}}$
**Ba1**	4*f*	1/3	2/3	0.59432(3)	1.000	0.00960(9)
**Ba2**	2*b*	0	0	1/4	1.000	0.00889(10)
**Nb1**	2*a*	0	0	0	0.887(4)	0.00409(16)
**Nb2**	4*f*	1/3	2/3	0.15720(2)	0.132(4)	0.00448(6)
**Ir1**	4*f*	1/3	2/3	0.15720(2)	0.868(4)	0.00448(6)
**Ir2**	2*a*	0	0	0	0.113(4)	0.00409(16)
**O1**	6*h*	0.48600(4)	0.97200(4)	1/4	1.000	0.0076(5)
**O2**	12*k*	0.16330(3)	0.32670(6)	0.07864(19)	1.000	0.0110(4)

Fig. S1. EDS measurement results. 2 different sites and 8 spectrums on each site were measured.

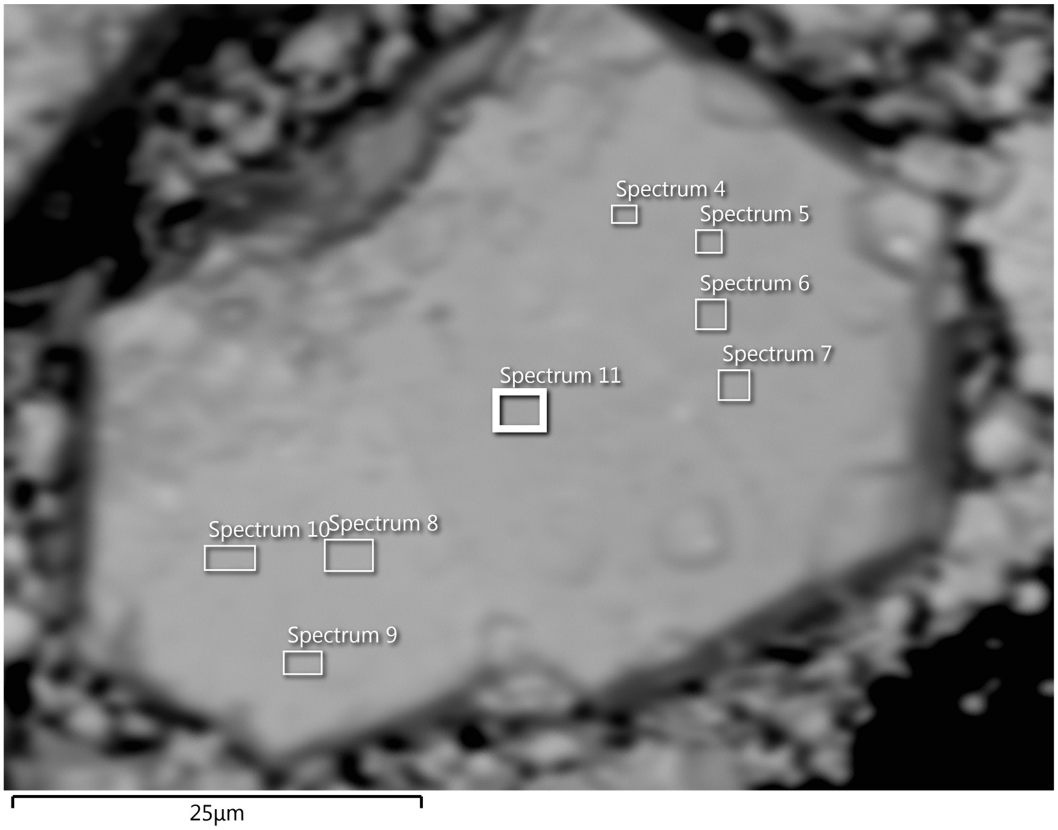

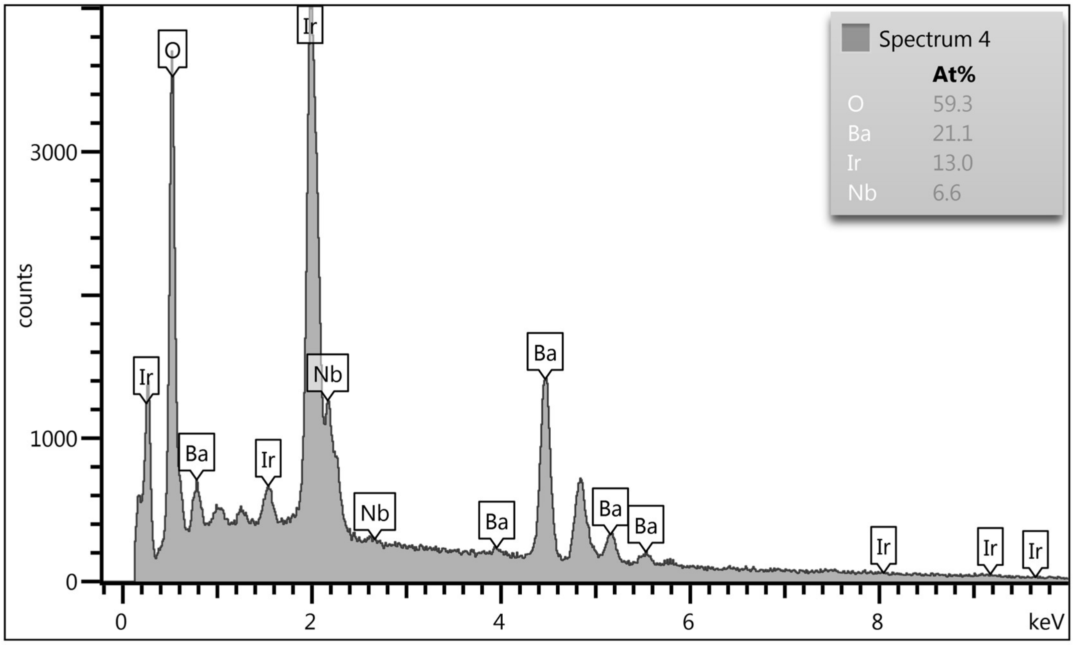

Fig. S2. Temperature-dependent of specific heat under 0 T (black) and 9 T (red). No significant differences were observed.

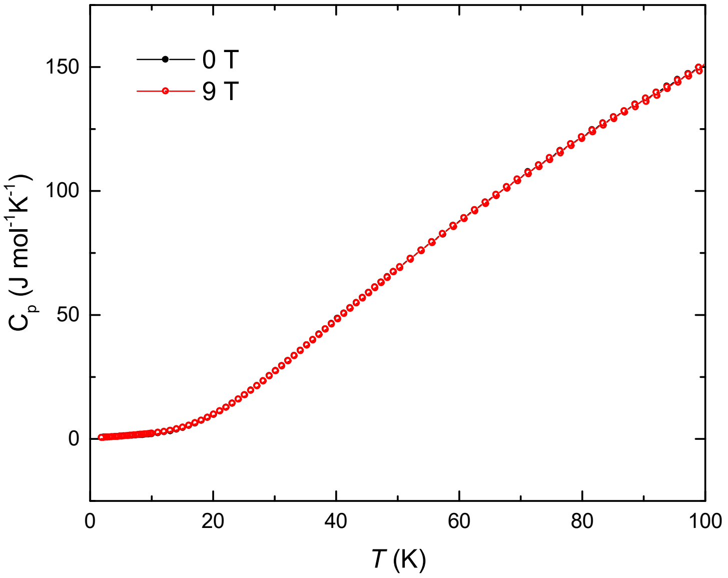

Fig. S3. Low-temperature heat capacity plotted as *C*p/T vs. T^2.

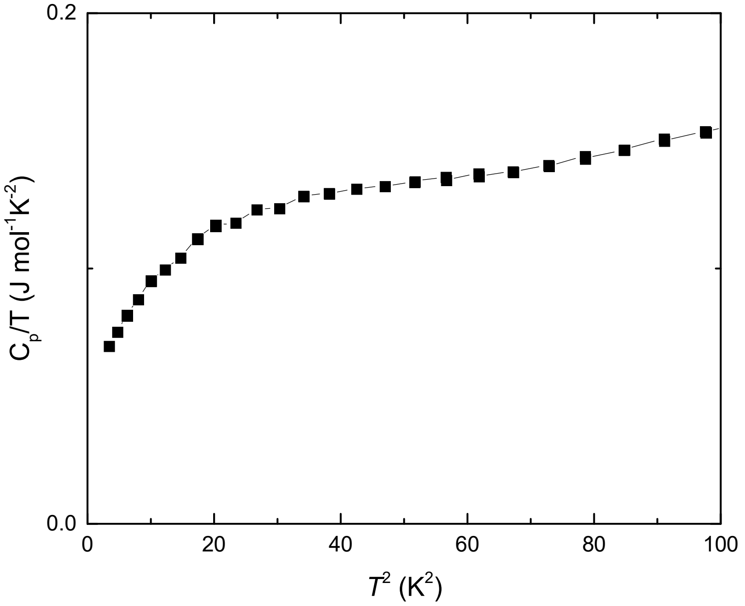

Fig. S4. Specific heat data fitted by (a) Debye and (b) Einstein model. The Debye and Einstein temperatures are determined as 427(4) and 304(4) K, respectively.

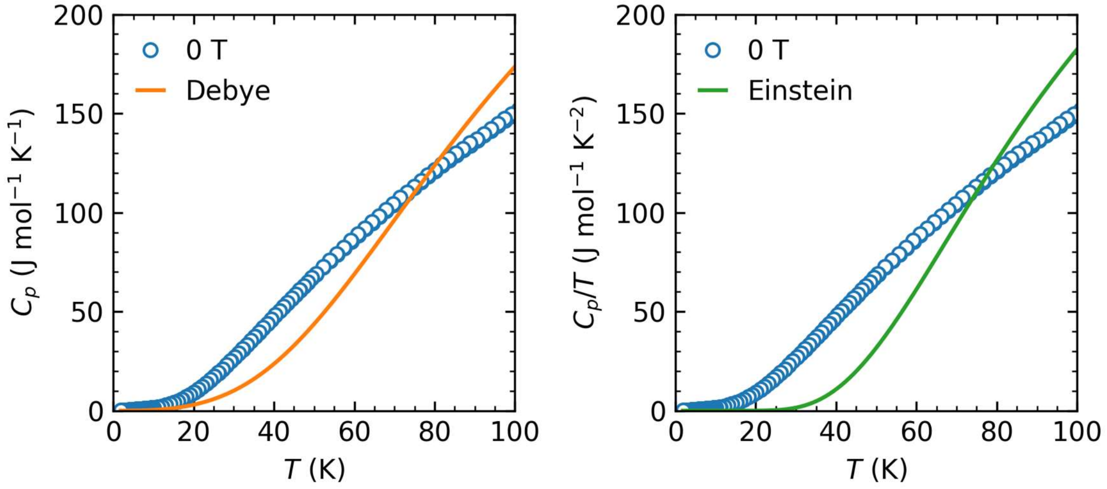

Fig. S5. Temperature-dependent resistance fitted at low temperature range

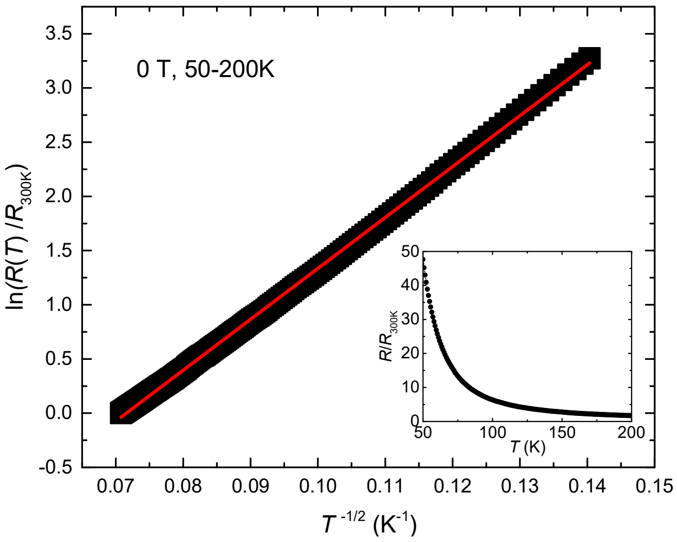

Supplement: Supplementary file 1 [file ic5c04558_si_001.pdf]
